# Health care providers’ perceptions of and attitudes towards induced abortions in sub-Saharan Africa and Southeast Asia: a systematic literature review of qualitative and quantitative data

**DOI:** 10.1186/s12889-015-1502-2

**Published:** 2015-02-12

**Authors:** Ulrika Rehnström Loi, Kristina Gemzell-Danielsson, Elisabeth Faxelid, Marie Klingberg-Allvin

**Affiliations:** Department of Public Health Sciences/IHCAR, Karolinska Institutet, Stockholm, Sweden; Department of Women’s and Children’s Health, Karolinska Institutet/Karolinska University Hospital Stockholm, Stockholm, Sweden; School of Education, Health and Social studies, Dalarna University, Falun, Sweden

**Keywords:** Induced abortion, Providers’ attitudes, Sub-Saharan Africa, Southeast Asia, Systematic review, Thematic analysis

## Abstract

**Background:**

Unsafe abortions are a serious public health problem and a major human rights issue. In low-income countries, where restrictive abortion laws are common, safe abortion care is not always available to women in need. Health care providers have an important role in the provision of abortion services. However, the shortage of health care providers in low-income countries is critical and exacerbated by the unwillingness of some health care providers to provide abortion services. The aim of this study was to identify, summarise and synthesise available research addressing health care providers’ perceptions of and attitudes towards induced abortions in sub-Saharan Africa and Southeast Asia.

**Methods:**

A systematic literature search of three databases was conducted in November 2014, as well as a manual search of reference lists. The selection criteria included quantitative and qualitative research studies written in English, regardless of the year of publication, exploring health care providers’ perceptions of and attitudes towards induced abortions in sub-Saharan Africa and Southeast Asia. The quality of all articles that met the inclusion criteria was assessed. The studies were critically appraised, and thematic analysis was used to synthesise the data.

**Results:**

Thirty-six studies, published during 1977 and 2014, including data from 15 different countries, met the inclusion criteria. Nine key themes were identified as influencing the health care providers’ attitudes towards induced abortions: 1) human rights, 2) gender, 3) religion, 4) access, 5) unpreparedness, 6) quality of life, 7) ambivalence 8) quality of care and 9) stigma and victimisation.

**Conclusions:**

Health care providers in sub-Saharan Africa and Southeast Asia have moral-, social- and gender-based reservations about induced abortion. These reservations influence attitudes towards induced abortions and subsequently affect the relationship between the health care provider and the pregnant woman who wishes to have an abortion. A values clarification exercise among abortion care providers is needed.

**Electronic supplementary material:**

The online version of this article (doi:10.1186/s12889-015-1502-2) contains supplementary material, which is available to authorized users.

## Background

Unsafe abortions are directly correlated with poverty, social inequity and the constant, methodical denial of women’s’ human rights [1]. The United Nations Committee on the Elimination of Discrimination against Women argue that women alone have the right to decide whether to have an abortion [2].

The denial of a pregnant women’s right to independently make this decision violates or poses a threat to a number of human rights, including a woman’s right to equality, liberty, non-discrimination, privacy, health and to be free from inhumane and degrading treatment, as explicitly articulated by the United Nations [2,3].

Unsafe abortions are a public health burden mainly in low-resource settings, with the highest burden in sub-Saharan Africa, Latin America and the Caribbean, followed by South and Southeast Asia [4]. At the opposite extreme, the rate of unsafe abortions in Europe and North America is insignificant [4]. A systematic literature review by Khan et al. [5] found that the maternal abortion-associated mortality ratio was 37 deaths per 100.000 live births in sub-Saharan Africa, 23 per 100.000 in Latin America and the Caribbean and 12 per 100.000 in South Asia. In countries with legal access to safe abortion services, deaths related to abortion are virtually non-existent [5]. According to the most recent global estimates for abortion-related deaths by WHO, unsafe abortions are responsible for approximately 47,000 deaths each year [6]. Kassebaum et al. indicated that abortion-related maternal deaths significantly decreased during 1990 to 2013 at a global level, except for sub-Saharan Africa where they significantly increased [7].

Induced abortions are legal on various grounds in several sub-Saharan Africa and Southeast Asian countries [8]. However, the health care providers in these countries often persist in viewing induced abortion as immoral, rather than recognising the legal status of abortion in their country [8].

In most high-resource countries, abortion laws were liberalised between 1950 and 1985 on safety and human rights grounds [9]. The most liberal abortion laws permit an abortion at the request of the women. However, there are vast differences in the abortion laws of different countries [10]. The United Nations has identified seven grounds on which an abortion is permitted: (1) to protect life of the mother, (2) to preserve the mother’s physical health; (3) to preserve the mother’s mental health; (4) in cases of rape or incest; (5) for foetal defects; (6) for socioeconomic reasons and (7) on request [10]. In most countries in sub-Saharan Africa and Southeast Asia, abortion laws are restrictive [10]. An abortion is legal at the request of the women in only two countries in sub-Saharan Africa: Cape Verde and South Africa. Cambodia, Singapore and Vietnam permit an abortion on a broad range of grounds [10]. In the last decade, several nations in these regions have liberalised their abortion laws to reduce the incidence of unsafe abortions. In 2005, Ethiopia approved a liberalised abortion law [11], and Ghana’s abortion laws have been fairly liberal since 1985. However, safe, legal abortion has not been well implemented until recent years and unsafe abortions are common in Ghana [12], and complications of induced abortions are the second leading cause of maternal death [13].

Women, particularly adolescent women and those who are poor and/or living in rural areas, often lack information about the legal status of abortions in their country and where to seek safe abortion services. In addition, they may lack the decision-making power and money to seek such services, or they might be discouraged by health care providers’ negative attitudes and a lack of confidentiality and privacy [14]. In many societies, abortion is a highly explosive topic, with stigma attached [15]. The latter may prevent women from accessing safe abortion services.

In many low-resource countries, the stigma associated with abortions means that the providers offering these services suffer discrimination in and outside the workplace [16,17]. The discrimination causes many providers to cease providing abortion services [16,17]. Furthermore, abortion providers’ attitudes may be in conflict with the national abortion law [18,19]. These conflicts may cause moral distress and hamper the professional–patient relationship. The lack of willingness and commitment among health care providers to deliver timely, thoughtful and supportive abortion care may directly or indirectly contribute to maternal mortality due to unsafe abortions. Therefore, it is important to understand health care providers’ perceptions of and attitudes towards induced abortions, as they have a substantial effect on the accessibility to abortion services and the quality of these services.

The aim of this systematic literature review was to identify, summarise and synthesise available research addressing health care providers perceptions of and attitudes towards induced abortions in sub-Saharan Africa and Southeast Asia.

## Methods

### Study identification

A comprehensive literature search of three databases (PubMed, CINHAL and Web of Science) and a manual search of reference lists of the identified studies were undertaken. All the searches took place during November 2014 and included all peer-reviewed articles, regardless of the year of publication (1977 – 2014). Each database was searched systematically for relevant citations using a high-sensitivity and low-specificity approach, as follows.

The systematic search was segregated into three elements: 1) health care provider, 2) abortion and 3) sub-Saharan Africa/Southeast Asia. For each of the elements, a list of relevant medical subject headings (MeSH), free text words, synonyms, abbreviations and alternate spellings that the authors might have used was accumulated. All the MeSH words and free text words were then combined using ‘OR’ to provide a large range of studies for each element. The three lists for each of the elements were then combined with ‘AND’ to generate high-sensitivity and low-specificity citations that were relevant to all three elements of the research question. The reference lists of the retrieved articles were screened to identify further relevant papers.

### Inclusion criteria

We decided to limit our review to sub-Saharan African and Southeast Asian countries, where the burden of maternal mortality is high. A thorough global analysis of health care providers’ attitudes towards abortion in other settings is beyond the scope of this paper and deserves attention in its own right.

The inclusion criteria for this literature review were: all primary quantitative and qualitative research studies that used data collection methods, such as surveys, self-completed questionnaires, in-depth interviews, focus-group discussions and observations to explore health care providers’ and students’ attitudes towards and perceptions of induced abortion in sub-Saharan Africa and Southeast Asia. The publications had to be written in English and pass a quality check developed by the first author (see Table 1).

**Table 1:** Quality assessment criteria*

	**Criteria**
**Aims**	General aims, specific objectives or research question clearly described.
**Background**	A comprehensive literature review included. Explanation and justification for the study.
**Context**	Context of the research adequately described.
**Sampling/recruitment**	Clear description of the sample, including the size and characteristics of the sample Selection procedure appropriate and clearly described Study units are representative of the target population Exclusions and refusals accounted for and described
**Data collection**	Suitable research design to address the aims of the research. Appropriate data collection instruments are used, piloted, pretested and described Clear description of the research methodology used Researcher - participation relationship adequately considered Ethical issues considered
**Data analysis**	Clear description of the data analysis method, process and findings.
**Data interpretation**	Clear discussion of the research findings. The study presents sufficient original data to support the findings, and to demonstrate that these and the conclusions are grounded in the data Clear integration of the data, interpretation and conclusions Study context and sample considered in the findings
**Reliability/validity**	Reliability and validity of the analysis has been addressed Rigorous data analysis

*Adapted from the Critical Appraisal Skills Programme (CASP) within the Public Health Resource Unit (PHRU) and existing instruments for studies on reproductive health [18,19].

### Selection of studies

**Figure 1:**
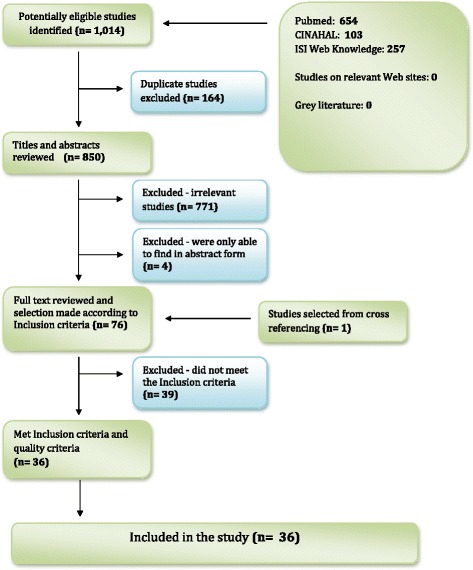
**Flow chart for identifying relevant studies.**

The first author selected and collected the articles for the review. This procedure included four steps (Figure 1). First, all 1,014 potentially relevant records identified from the electronic searches underwent an initial title and abstract review to determine their relevance according to the inclusion criteria. These records were then imported into bibliographic software EndNote® for reference management. The EndNote® library was later searched to identify duplicate files. All duplicates were deleted, and a single copy of each record was retained. Second, a hard copy of all the potential studies was obtained and analysed according to the inclusion criteria. Studies that did not meet the inclusion criteria were excluded. Third, all articles that met the inclusion criteria were reviewed to determine the quality of the study (see Table 1). Studies that did not meet the pre-set methodological quality criteria, described below, were excluded. This process is clarified under the next section. Finally, the reference lists in the retrieved articles were screened to identify further relevant papers according to the inclusion and quality criteria. Figure 1 describes the reasons for the exclusion of studies.

### Assessment of the quality of the study and data extraction

To assess the methodological quality (internal and external validity) of the included studies, the main author developed a checklist of quality criteria based on the critical appraisal skills programme and existing instruments for studies on reproductive health [19,20]. The following eight criteria were assessed: 1) aim, 2) background, 3) context, 4) sampling, 5) data collection, 6) data analysis, 7) data interpretation and 8) reliability/validity. Table 1 provides a detailed description of the quality criteria.

Following the initial reading of the 36 included studies; each study was read several times by the main author to appraise the content. Its findings were then summarised on a data extraction form by the main author. The following information was recorded: background of the study, country of research, study population, study characteristics, design and methods, methodological quality, data sampling, data collection, analysis methods and key findings.

### Synthesis

In the review, both qualitative and quantitative data were assessed, and each study was analysed individually. The results from the selected studies were analysed using a thematic analysis that was used previously for synthesising results in systematic literature reviews of qualitative and quantitative studies [20-22]. The 36 studies included in this review were first assessed to categorise key descriptive themes. The key descriptive themes were then systematised in a matrix, and similarities, differences and contradictions were examined. To answer the review question about the attitudes of health care professionals towards induced abortions in sub-Saharan Africa and Southeast Asia, analytical themes were created.

## Results

### Description of the studies included in the review

The 36 studies included in the review were published between 1977 and 2014 and described health care professionals’ perceptions of and attitudes towards induced abortions. The studies were conducted in 15 countries: Ethiopia (1), Ghana (5), Indonesia (1), Kenya (1), Mozambique (1), Myanmar (1), Nigeria (4), South Africa (13), Swaziland (1), Thailand (2), Timor Leste (1), Uganda (1), Vietnam (2), Zambia together with Kenya (1) and Zimbabwe (1). In total, 29 studies were conducted in sub-Saharan Africa, and seven took place in Southeast Asia. Table 2 describes the characteristics of the studies included in the review.

**Table 2:** **Characteristics of the studies included in the review**

**Nr.**	**Country**	**Year of publication**	**Study period**	**Abortion law** ^*^	**Aim**	**Sample size/characteristics**	**Data collection**	**Reference**
**1.**	Ethiopia	2011	March – April 2008	A B D E H +	To answ er the questions; “what does perceptions on safe abortion look like among health care service providers?” “What are the factors which affect the perception of health providers towards safe abortion?”	431 health providers	A structured, self-administered questionnaire	ABDI, J. et al. [32]
**2.**	Ghana	2013	No information	A B C D E H	To examine in in what ways provider attitudes and values affect the implementation of abortion policy.	43 health professionals	In-depth interviews	ANITEYE, P. et al. [30]
**3.**	Timor Leste	2009	2006 -2007	A	To describe the socio-legal context of unsafe abortion in Timor-Leste	21 doctors and midwives	In-depth interviews	BELTON, S. et al. [24]
**4.**	South Africa	2000	No Information	A B C D E F G -1st H	To investigate health care ethics regarding Termination of pregnancy	1200 registered nurses	Questionnaires and Focus-group discussions	BOTES, A. [16]
**5.**	South Africa	2002	No Information	A B C D E F G -1st H	To assess attitudes of medical students to induced abortion	247 medical students	Self-administered questionnaire	BUGA, G.A. [33]
**6.**	South Africa	2005	Nov. 2001 – March 2002	A B C D E F G -1st H	To explore attitudes of health care providers towards medical abortion	20 public health nurses and doctors	In-depth interviews	COOPER, D., et al. [25]
**7.**	Indonesia	1993	Oct. 1990 – April 1991	A	To contribute to the search for ways to make pregnancy and childbirth safer	28 Physicians, 16 Midwives,16 TBA 16 PLKB Total: 76	In-depth interviews	DJOHAN, E., et al. [26]
**8.**	Nigeria	2003	No Information	A	To examine the knowledge, attitude and practice of private medical practitioners on abortion	48 private practitioners	Structured questionnaire	ETUK, S.J. et al. [34]
**9.**	Mozambique	2004	2002	A B	To document the strengths and deficiencies of abortion care	99 Midwives and nurses	Questionnaire	GALLO, M.F., et al. [35]
**10.**	South Africa	2000	No Information	A B C D E F G - 1st H	To explore and describe nurses’ experiences of being involved with abortion care	Nurses	Phenomenological interviews	GMEINER, A.C., et al. [31]
**11.**	South Africa	2012	July – October 2008	A B C D E F G - 1st H	To explore health service providers’ perceptions of abortion services	19 providers and hospital managers	In-depth interviews	HARRIES, J. et al. [27]
**12.**	South Africa	2009	2006 - 2007	A B C D E F G - 1st H	To explore knowledge, attitudes and opinions of health care providers’ attitude to abortion	34 health care providers	In-depth interviews and one focus group discussion	HARRIES, J. et al. [53]
**13.**	South Africa	2000	No Information	A B C D E F G - 1st	To study attitudes and beliefs about abortion among nurses	24 male and 114 female nurses In total 138	Self administered questionnaire	HARRISON, A., et al. [36]
**14.**	Zimbabwe	1999	No Information	A B D E H	To determinate the attitudes of professional health workers to medically supervised abortion	196 doctors, 1 053 nurses In total 1249	Self administered anonymous questionnaire	KASULE, J., et al. [37]
**15.**	Kenya	1992	April 1991	A	To determine nurses’ knowledge and attitudes towards abortion	218 nurses	Self-administered questionnaire	KIDULA, N. A., et al. [38]
**16.**	Vietnam	2006	March – April 2002	A B C D E F G	To explore the midwives’ perspectives on adolescent sexuality and abortion, and what they consider to be quality abortion care	40 Midwives; 28 doctors In total 68	Observations and focus-group discussions (FGD)	KLINGBERG-ALLVIN, M., et al. [54]
**17.**	Vietnam	2007	2003	A B C D E F G H	To investigate midwifery students’ values and attitudes towards adolescent sexuality, abortion and contraception	235 midwifery students	Quantitative survey complemented with 18 qualitative interviews	KLINGBERG-ALLVIN, M., et al. [55]
**18.**	South Africa	2005	No Information	A B C D E F G - 1st H	To explore the lived experience of midwives who assist with TOP	3 nurses	In-depth interviews	MAYERS, P.M. et al. [28]
**19.**	Swaziland	2008	January – March 2005	A B C D E H	To explore health workers’ perceptions of adolescent SRH services in Swaziland	56 midwives	Self-administered questionnaire	MNGADI, P. T., et al. [39]
**20.**	South Africa	2008	No information	A B C D E F G 1^s t^H	To investigate professional nurses’ attitudes towards abortion care	25 nurses	Questionnaire	MOKGETHI, N. E. et al. [40]
**21.**	Ghana	2007	August 2003	A B C D E H	To assess physician’s knowledge and attitude towards abortion	74 physicians	Self-administered questionnaire	MORHE, E.S. et al. [41]
**22.**	Nigeria	2005	No information	A	To investigate the attitudes and practices of physicians towards abortion	232 private practitioners	Structured questionnaire	OKONOFUA, F. E. et al. [42]
**23.**	Nigeria	2011	No information	A	To assess the attitudes of staff at reproductive health services	136 senior practitioners	Questionnaire	OMO-AGHOJA, L.O. et al. [43]
**24.**	Nigeria	2009	27 December 2005 to 25 March 2006	A	To understand PAC services provided by private medical practitioners	96 private medical practitioners	Questionnaire	ONAH, H. E. et al. [44]
**25.**	Uganda	2014	February - March 2012	A	To explore physicians’ and midwives’ perceptions of PAC	10 Doctors and 17 Midwives In total 27	In-depth Interviews	PAUL, M., et al. [29]
**26.**	Ghana	2013	Fall 2009	A B C D E H	To explore the reasons women continue to die from unsafe abortion	4 Physicians	Open ended interviews and Focus-group discussion	PAYNE, M.C., et al. [51]
**27.**	Thailand	1986	1980 - 1981	A B C D E	To demonstrate health professionals’ attitudes toward abortion	625 Doctors, Nurses and social workers	Self-administered questionnaire	PHUAPRADIT, W., et al. [45]
**28.**	South Africa	1998	No information	A B C D E F G - 1st H	To explore and describe nurses’ experience of abortion	1200 nurses	Focus-group discussions, interviews and observations	POGGENPOEL, M., et al. [56]
**29.**	South Africa	2004	No information	A B C D E F G - 1st H	To compile a profile of the characteristics and/or beliefs held by nurses who choose to become abortion providers	22 nurses	Focus-group discussion and deep-interviews	POTGIETER, C [57]
**30.**	Ghana	2013	March – April 2008	A B C D E H	To understand pathways to induced abortion in Ghana and the role health care providers play	11 Family planning nurses and 8 obstetricians/gynaecologists In total 19	Focus-group discussion and in-depth interviews	SCHWANDT, H.M., et al. [51]
**31.**	Myanmar	2012	March – May 2011	A	To find out medical students’ knowledge of and attitudes toward abortion	1,060 Medical students	Questionnaire	TEY, N.P., et al. [50]
**32.**	Thailand	1977	No information	A B C D E	To evaluate the attitudes of medical students towards abortion	318 medical students	Questionnaires	VARAKAMIN, S. et al. [46]
**33.**	Ghana	2010	February 2007	A B C D E H	To assess the capacity and willingness of midwifery tutors to teach abortion care	74 Midwifery tutors	Structured questionnaire	VOETAGBE, G. et al. [47]
**34.**	South Africa	1995	No information	A B C D E F G - 1st H	To explore the understandings and responses of nurses towards abortion	35 Primary Health Care Nurses	Observations and interviews	WALKER, L. [58]
**35.**	Kenya and Zambia	2006	Sept. – Dec. 2001	Kenya A Zambia A B C E F	To investigate attitudes among Kenyan and Zambian nurse-midwives toward adolescent SRH problems, in order to improve services for adolescents.	322 Nurses from Kenya 385 Nurses from Zambia In total 707 Nurses	Questionnaires	WARENIUS, L. U., et al. [48]
**36.**	South Africa	2012	2005 - 2007	A B C D E F G - 1st H	To assess attitudes about abortion provision and future practice intentions of medical students	1308 medical students	Self-administered questionnaire	WHEELER, S.B. et al. [49]

A = To protect woman’s life D = Rape G = On request 1st - First trimester only.

B = Physical health E = Foetal defects H = Incest.

C = Mental health F = Socio-economic factors.

+ = Abortion permitted on additional enumerated grounds relating to such factors as the woman’s age or capacity to care for a child.

*Source: CENTER FOR REPRODUCTIVE RIGHTS (2009) World Abortion Laws 2009 Fact Sheet.

http://reproductiverights.org/sites/crr.civicactions.net/files/documents/pub_fac_abortionlaws2009_WEB.pdf.

According to the World Bank’s analytical income categories [23], the studies were conducted in one upper middle-income country, seven lower middle-income countries and five low-income countries.

Seven of the studies [24-30] used in-depth interviews as the method of data collection, one study used a phenomenological approach to interviews [31], and 19 of the studies used self-completed questionnaires [32-50]. Nine of the studies [16,51-58] had used more than one data collection method, such as surveys, observations, focus group discussions and in-depth-interviews.

The health care providers’ attitudes towards induced abortions were classified into nine key descriptive themes: 1) human rights, 2) gender, 3) religion, 4) access, 5) unpreparedness, 6) quality of life, 7) ambivalence, 8) quality of care and 9) stigma and victimisation. These nine key descriptive themes were collapsed into five analytical themes based on their content.

### Human rights and quality of life

The synthesis revealed that health care providers, in general, were uncertain about the legal status of abortion in their countries [24-26,32,37,41,43,47,53]. Some health care providers considered induced abortion as a significant public health problem and perceived the legalisation of abortion as a positive step because, otherwise, women would opt for an unsafe abortion, risking their lives [26,29-32,37,47,49,57]. However, nurses and midwives from South Africa, which has a more liberal abortion law, concluded that if women had the right to make choices regarding the termination of their pregnancy, health personnel should have the right to choose whether to work in abortion clinics [27,36,53,56].

The health care personnel who participated in the studies conducted in South Africa and Vietnam considered that an increase in urbanisation and improvements in access to education had changed the context of sexual and reproductive behaviour [27,36,53,54]. As a result, they believed that women and adolescents were in need of sexual and reproductive health information, including family planning, to prevent unwanted pregnancies and induced abortions [27,36,53,54].

According to the results, the majority of health care providers were supportive of abortions in cases where the pregnancy was due to rape or incest, severe genetic disorders were present, or it was necessary to save the life of the woman [24,26-30,35,36,38,40,45,46,49,53]. Only one study included in this literature review explored nurses’ attitudes towards abortions among women living with HIV/AIDS [40]. In this South African study, the respondents suggested that these women should have access to abortion services.

Regardless of the liberal abortion laws in South Africa, the nurses and midwives stated that the foetus should also have rights [16,36,40]. They asserted that national hospitals were established to save lives, not to eliminate them [16,36,40]. In addition, the nurses disapproved of childless women having an abortion [16,36,40].

A South African study revealed that nurses who had been trained in abortion care considered a woman’s access to an induced abortion as a human right [57]. They felt that their training would enable them to reduce the maternal mortality and morbidity caused by unsafe abortions [57]. A recent study among medical students in South Africa found that 70 per cent of the respondents believed that it is the right of the woman to decide whether to have an abortion [49]. In one study from Ghana, it was found that human rights arguments were used both for and against abortion care [30].

### Gender, stigma and victimisation

In three of the studies, the nurses and midwives stated that women should give birth and care for their children and expressed the view that induced abortion was ‘*terminating motherhood*’ [17,26,28]. In the same studies, these nurses and midwives considered that women who choose an abortion denied their role as mothers and thus rejected their identity as women.

Three studies conducted in Southeast Asia (Indonesia and Thailand) reported differences between the sexes regarding attitudes towards induced abortion, with female health care providers apparently having more conservative attitudes than male personnel [26,45,46]. Only one study from Thailand reported differences in attitudes towards abortion in relation to the respondent’s age [45]. This study suggested that the attitudes of younger nurses towards abortions were more liberal than those of older nurses.

In several studies, abortion providers mentioned they were perceived by their colleagues as murderers or ‘*baby killers’* [16,25,28,33,36,40,54,56-58]. In two studies from South Africa, the providers considered that an abortion was a threat to the community, an act of disgrace and a waste of taxpayers’ money [16,58].

However, some nurses from South Africa indicated that they would assist a woman who suffered complications following an unsafe abortion. They considered that the stigma associated with the induced abortion would be attached to the individual who performed the abortion procedure, rather than the nurse who was only fulfilling her professional duty in saving a woman’s life [36,56]. At the same time, they verbalised that they had chosen nursing because they wanted to preserve life and promote health, not act as murderers [36,56]. Gynaecologists and general practitioners participating in an Indonesian study articulated that the life of a foetus commences after 120 days of pregnancy [26]. Therefore, they did not consider ‘*menstrual regulation’* as a form of abortion, and it was thus widely accepted [26].

In both sub-Saharan Africa and Southeast Asia, the health care providers experienced personal conflicts, stigmatisation and victimisation because of the negative attitudes of family, community, fellow health care workers and policymakers [18,26,28-30,40,51,52]. Colleagues and friends rejected them and voiced negative comments, for example, calling them ‘*killers*’ [16,25,27,28,40].

Moreover, the synthesis emphasised that nurses in Southeast Asia and South Africa strongly disapproved of pre-marital sex, although this was described as a modern trend among the young and unmarried [26,36,39,52,54,55]. Nevertheless, pregnancy outside of marriage was not accepted [26,36,39,54,55]. Nurses in Vietnam considered that not having pre-marital sex was the best solution to reduce abortion rates among unmarried young women [54,55].

### Religion

Eighteen studies from sub-Saharan Africa and Southeast Asia identified religion as the most important factor influencing the attitudes of health care providers towards induced abortions. [16,24,26,28,30-34,36,37,40,43,44,47,56-58]. The respondents in those studies believed that only God can decide between life and death and that abortion was a sin. However, in a recent study from South Africa, the nurses viewed abortions differently, depending on whether they were medical or surgical [25]. They perceived that a medical abortion was in the hands of the woman and therefore the woman, not the nurse, had to answer to God for her actions [25].

### Unpreparedness and ambivalence

In the majority of the studies, the health care providers and students revealed that they felt unqualified and unprepared for work in the area of induced abortions [26,28,29,31,34-41,44,45,47,48,50,53-58]. In addition, they reported a lack of standard care guidelines and support and highlighted the need for cognitive, emotional and spiritual support [26,28,31,34-41,44,45,47,48,53-58].

Nurses from South Africa considered abortion to be against the nurses’ professional code, which requires them to save lives [36]. The nurses expressed ambivalence between their professional responsibilities and personal norms and values. They were angry with the patients who requested induced abortions, blaming them for destroying the nurse’s pledge to be a caregiver [16,28,36,56].

### Access and quality of care

In general, the nurses and midwives disliked being involved with abortion services, and they commonly reported hesitance in providing these services [16,28,38,40,48,53,55,56,58]. Midwifery students from Vietnam revealed that the main reason for choosing midwifery as a profession was to care for women in labour and delivery, and hardly any of the students wanted to work in the area of abortion services [55]. Similar attitudes were reported among physicians [53]. Furthermore, managers in two studies from South Africa expressed difficulties when recruiting, retaining and scheduling health care providers for induced abortion procedures [28,53]. Three other studies from South Africa concluded that nurses’ resistance to providing abortion services was a powerful barrier against access safe abortion services, with nurses’ and midwives’ strong opposition to abortion affecting rural women in particular [16,25,36].

Several studies from sub-Saharan Africa showed that nurses and midwives have judgmental attitudes towards abortion patients [31,36,40,58]. In general, the nurses seemed to withdraw from the patients and ignored their responsibilities as caregivers [16,31,39,40,54]. Furthermore, respondents from both sub-Saharan Africa and Southeast Asia said they could not provide holistic nursing care to women undergoing an induced abortion because they had negative feelings about the woman’s decision [36,55]. The nurses and midwives also recognised that these women received inadequate care due to the poor relationship between the nurse and the patient [28,39,40,55,56].

On the other hand, a study by Cooper et al. [25] gave a positive view on nurses’ and midwives’ attitudes towards abortion. In this study, the nurses expressed a strong interest in medical abortions. In other studies, health care providers, in general, preferred medical abortions, as these required minimal involvement on their part in the abortion process [25,27]. Furthermore, early termination of pregnancy (i.e. menstrual regulation) was more accepted among health care providers than second-trimester abortions [26,27,46].

## Discussion

### Main findings

In total, 36 studies with qualitative or quantitative data from 15 different countries met the inclusion criteria. A thematic analysis of the data indicated that health care providers in sub-Saharan Africa and Southeast Asia have negative feelings about induced abortions.

### Strengths

To our knowledge, this is the first systematic review to evaluate health care providers’ perceptions of and attitudes towards induced abortions in sub-Saharan Africa and Southeast Asia. The data are based on the individual participant’s perspective of induced abortions.

### Limitations

It is critical to note that the review is limited to 15 countries: 10 in sub-Saharan Africa and five in Southeast Asia. Although the key themes were common across most of the studies, we do not suggest that health care providers’ perceptions of and attitudes towards induced abortions will be homogenous in all countries in sub-Saharan Africa and Southeast Asia. Non of the studies that we reviewed measured the effect of health care providers’ perceptions of and attitudes towards induced abortions on access to safe, high-quality abortion care.

Further limitations of the review include the following: 1) 36 percent of the studies were from South Africa, making it difficult to generalise the findings to other populations or settings; 2) only one article reported provider attitudes in Ethiopia, which is the second largest country in sub-Saharan Africa and the abortion law was liberalized almost ten years ago and legal, induced abortion services have rolled out nationwide since then. Provider attitudes might be more positive toward abortion and abortion care in Ethiopia than in other countries in the region. 3) The method of data collection was different in each study, and the research question varied between the studies; 4) none of the studies used questions/statements in a Likert format when assessing the perceptions’ of or attitudes towards induced abortions, and they were thus not able to capture the intensity of the providers’ feelings about induced abortions, 5) most of the studies asked for provider’s opinions on induced abortions rather than directly measuring practice, 6) many studies did not consider other influencing factors, such as sex, age and further education or training, 7) the sample selection of the respondents differed according to the study, and 8) the articles were published during a long period (1977–2014), and attitudes towards abortions might have changed in the two regions during this time. Thus, caution is needed with regard to generalisation of the results.

### Interpretations

This systematic literature review demonstrated that health care providers in sub-Saharan Africa and Southeast Asia have conservative attitudes towards induced abortions. These attitudes were manifested in a judgmental approach towards women with unwanted pregnancies who requested an induced abortion. The health care providers described how these women were ignored and treated with a lack of respect.

In general, the participants viewed an induced abortion as ending a human life and considered it a mortal sin. Religious beliefs affected these views. However, many providers considered that menstrual regulation was acceptable and did not view it as an abortion. Likewise, nurses in South Africa perceived medical abortion as different from surgical abortion, with the former widely accepted as being in the hands of the women who had to answer to God for their actions, not the nurse. In cases where the pregnancy was due to rape or incest, the health care providers seemed to have more sympathy for the woman, and they did not blame the abortion on her not using contraceptives.

One important finding of this review was that some of the nurses and midwives considered that the expectation to provide an induced abortion conflicted with their professional duty to protect life, based on the Code of Ethics for Nurses. Many of the nurses cited this code and highlighted how it contradicted the provision of abortion care services and thus created feelings of guilt, ambivalence and anxiety among nurses.

Midwives mentioned that they were trained to assist women in labour and delivery, not to assist during the termination of a pregnancy. Both the “Essential Competencies for Basic Midwifery Practices 2010” and the new ICM model for the midwifery curriculum by the International Confederation of Midwives include abortion care and family planning services [59,60]. However, a striking finding in this review was that the nurses and midwives seemed to be unprepared to care for women with unwanted pregnancies.

The World Health Organization recommends task shifting, which is a practice of delegation, whereby certain tasks are distributed from physicians to nurses and midwives [61]. In task shifting, the heath care workforce is used in a more efficient way, as the roles of health care workers are optimised [61]. Studies conducted in South Africa, Vietnam and Nepal showed that the provision of abortion care by midlevel health care providers is as safe and effective as the abortion care provided by physicians [62,63]. However, task shifting might be challenging to implement if nurses and midwives are reluctant to provide abortion care. This review emphasises that health care providers, in general, and nurses and midwives, in particular, need values clarification and technical training in comprehensive abortion care before they can commit to the responsibility of providing quality abortion care.

Access to safe, legal induced abortion, postabortion care (which occurs after an unsafe abortion) and family planning is fundamental to reduce maternal mortality and morbidity related to unsafe abortions [64,65]. The conservative attitudes towards induced abortions among health care providers in sub-Saharan Africa and Southeast Asia might also affect access to post-abortion care and, consequently, post-abortion contraceptive counselling.

It is essential to highlight that the majority of the studies included in this review were conducted in South Africa, where it is known that many health care providers are conscientious objectors to the provision of safe abortions [17,66]. The refusal to assist in abortion services is frequently based on moral, religious, ethical or philosophical beliefs. As reported elsewhere, such conscientious objections to abortion provision are an abuse of women’s rights and potentially harmful to women’s health [67]. A recent study from Ghana indicates that a favourable attitude toward abortion among health care providers’ is not associated with safe abortion provision. On the other hand, it was noticed that the odds of providing safe abortions lowers by 57 percent when the health care provider is Catholic in comparison to other religions. Furthermore, the same study found that providers’ confidence in their capability to offer safe abortion is fundamental [68].

Abortion care providers need to be prepared, supported and assisted [67,68]. The ethical dilemmas of reproductive health care providers charged with providing abortion services require more attention in pre-service and in-service training programmes. The pre-graduation curricula for health care providers in sub-Saharan Africa and Southeast Asia should include training in comprehensive abortion care, such as technical skills, interpersonal skills, value clarification, and communication and counselling in family planning and abortion [69]. Some researchers have argued that value clarification, together with supportive follow-up, may have a positive impact on health care workers’ attitudes [70,71].

The impact of health care providers’ attitudes on access to abortion care and the availability of quality abortion care, in addition to what extent value clarification can positively influence these attitudes, remains to be studied. Finally, strategies to decrease barriers to midwifery-led induced abortion and post-abortion care need to be evaluated to expand access to abortion care.

The findings of this review can be used to design interventions to increase women’s access to safe abortion care, post-abortion care and family planning services in specific regions.

## Conclusions

This systematic literature review suggests that religious convictions, beliefs about professional roles and ethics and feelings of unpreparedness frequently give rise to dilemmas among health care providers responsible for the provision of abortion care in sub-Saharan Africa and Southeast Asia. Moral-, social- and gender-based reservations about induced abortions appear to influence health care providers’ perceptions of and attitudes towards induced abortions and, consequently, their relationship with the patient who wants an abortion. Political commitments and resources are needed to ensure that health care providers are trained to develop the competencies to enable them to perform safe, high-quality abortions and to advocate for abortion care.

Furthermore, this review found that health care providers considered menstrual regulation and medical abortion more acceptable than manual vacuum aspiration. Hence, stakeholders and policy planners urgently need to introduce these two abortion methods, especially in rural health districts, to improve access to abortion services and, consequently, reduce maternal morbidity and mortality due to unsafe abortions. The findings from this review have implications for policy makers and hospital managers when organising health care services. Introducing value clarification and training in abortion care and services might increase the availability and accessibility of quality abortion care.

### Details of ethical approval

Ethical approval was not necessary because the literature review used secondary data from published articles.
